# A cell-penetrating bispecific antibody suppresses hepatitis B virus replication and secretion

**DOI:** 10.1016/j.virusres.2025.199531

**Published:** 2025-01-31

**Authors:** Chongwei Xie, Bing Zhou, Da Yao, Xin Wang, Lihong Zhong, Chuanghua Qiu, Junfang Zhang

**Affiliations:** a Medical Research Center, Yuebei People's Hospital, Shantou University Medical College, 512025, Shaoguan, China; b Shenzhen Immuthy Biotech Co., Ltd, 518107, Shenzhen, Guangdong, China; c Institute for Hepatology, National Clinical Research Center for Infectious Disease, Shenzhen Third People's Hospital, The Second Affiliated Hospital, School of Medicine, Southern University of Science and Technology, 518112, Shenzhen, Guangdong, China; d The First Affiliated Hospital of Shenzhen University, Shenzhen Second People's Hospital, 518037, Shenzhen, Guangdong, China

**Keywords:** Cell-penetrating bispecific antibody, HBcAg, Cell-penetrating peptide, HBV

## Abstract

Hepatitis B virus (HBV) represents one of the major pathogenic factor that leads to chronic liver diseases and the development of hepatocellular carcinoma (HCC). The currently approved anti-HBV drugs cannot eradicate the virus or block the development of HCC. HBV nucleocapsid consists of the hepatitis B core antigen (HBcAg) and the HBV relaxed-circular partially double-stranded DNA (rcDNA), indispensable in virus replication. The present study reported a cell-penetrating bispecific antibody targeting HBcAg and preS1, fused with the cell-penetrating peptide R9TAT, named Anti-preS1 × Anti-HBcAg-R9TAT. The antibody could recognize preS1 and HBcAg and internalize into living cells efficiently, suppressing the extracellular hepatitis B surface antigen (HBsAg) and hepatitis B envelope antigen, and the intracellular HBsAg and HBcAg *in vitro*. This cell-penetrating bispecific antibody is a novel approach to suppressing HBV replication and secretion and is a promising anti-HBV therapeutic antibody candidate.

## Highlights

• A cell-penetrating bispecific antibody Anti-preS1 × Anti-HBcAg-R9TAT could recognize HBV positive cells and target intracellular HBcAg.
• The cell-penetrating bispecific antibody suppresses hepatitis B virus replication and secretion.
• A novel approach to suppress HBV replication and secretion for anti-HBV therapeutic antibody.

## 1 Introduction

Hepatitis B virus (HBV) belongs to the hepadnaviridae family and is associated with liver diseases including hepatitis in humans (Liang, 2009). Chronic hepatitis is a high-risk factor in cirrhosis and hepatocellular carcinoma (HCC) development (Jeng et al., 2023). Every year >78,000 people die worldwide due to HBV infection (Loomba and Liang, 2017). The HBV vaccines have largely reduced new HBV infection cases; however, >300 million chronic HBV (CHB) carriers worldwide desire more effective therapy strategies (World Health Organization, 2019). The interferon and nucleos(t)ide analogs are a potent treatment for virological suppression, however, the treatments barely halt HCC development (Kang et al., 2015; Chien and Liaw, 2022). Various strategies targeting several key steps in the HBV life cycle have been studied, including repressing the infection to hepatocytes, interfering with virus transcription, blocking nucleocapsid assembly, inhibiting HBsAg release and disrupting or silencing HBV covalently closed circular (cccDNA). Among the clinical therapies for CHB, interferon therapy is currently the most effective; however, the disappearance of the hepatitis B envelope antigen (HBeAg) has been observed only in 20–30% of patients. The nucleos(t)ide analogs could benefit long-term HBV DNA level suppression (Zhang et al., 2021). It is urgently required to develop more effective drugs and treatments for HBV.

HBV is one of the smallest DNA virus, consisting of a lipid-protein envelope surrounding its icosahedral nucleocapsid (Li et al., 2020). The large (L) protein is mainly distributed on the surface of infectious viral particles with its preS1 region mediating the infection process. The nucleocapsid consists of the Hepatitis B core antigen (HBcAg), which is indispensable in HBV replication (Chi et al., 2009). The virus enters hepatocytes through the binding of preS1 to its receptor, the sodium taurocholate cotransporting polypeptide (NTCP) (Hatooka et al., 2022). HBeAg is processed from the pre-core protein, which is a secretory protein marker of replication and infectivity (Inoue and Tanaka, 2020).

This replication process is initiated by pre-genome RNA (pgRNA) formation, with the help of HBcAg and polymerase (Jiang and Hildt, 2020). The HBV nucleocapsid is made of HBcAg, the N-terminal domain is crucial for HBV assembly, while the C-terminal domain is responsible for viral pre-genomic RNA packaging (Lee and Tan, 2008). In the life cycle of HBV, HBV capsid has critical functions, such as reverse transcription, genome packaging and intracellular trafficking. HBcAg is a promising target for a new antiviral strategy and it has been shown that the capsid assembly modulators (CAMs) could block pgRNA encapsidation and HBV DNA replication by disturbing nucleocapsid formation (Nijampatnam and Liotta, 2019; Yuen et al., 2020; Yuen et al., 2019).

Most CHB patients produce a high-titer anti-HBcAg antibodies (De Conto et al., 2022), however they have no antiviral effect because the HBcAg is enveloped by HBsAg, the antibodies cannot approach it. Moreover, the antibodies cannot enter virus-infected hepatocytes. It was reported that delivering the DNA sequence of an anti-HBcAg antibody through a plasmid or lentiviral vector could inhibit HBV DNA replication (Peters and Locarnini, 2017). The gene therapies are challenged by transfection efficiency and safety concerns (Zhang et al., 2019; Klumpp et al., 2018; Yamamoto et al., 1999; Martinez et al., 2022), therefore, the investigators aimed to design a cell-penetrating antibody crossing the cell membrane and targeting the inner proteins. A possible solution was linking a cell-penetrating peptide (CPP) to the antibody. CPP are short peptide sequences, which can transport several types of cargos, accumulating within the inner cells (Guidotti et al., 2017). It has been reported that the anti-HBcAg transbody HBcMAb-TAT could penetrate the HepG2.2.15 cells and inhibit HBV replication (Wang et al., 2015). In a former study, we developed a cell-penetrating antibody 9D11-TAT targeting Hepatitis B virus X protein (HBx). HBx is a multi-functional protein of HBV and has an important role in initiating and maintaining virus replication (Zhang et al., 2018). The present results demonstrated that the 9D11-TAT could efficiently internalize into living cells and suppress HBV transcription, replication and virus protein production both *in vitro* and *in vivo* models.

CPP has no targeting character, CPP could take the antibodies to other cells which may induce off-target and safety concerns (Asrorov et al., 2023). To improve targeting treatment, we choose preS1 as the cell surface marker because it surrounds the hepatocyte's cell membrane surface when infected by HBV. The designed a cell-penetrating bispecific antibody is composed of Anti-preS1 and Anti-HBcAg fused with R9TAT, we found that the present bispecific antibody could internalize into the HBV-positive cells and target the inner HBcAg, it could not only recognize preS1 and HBcAg but also efficiently lower the extracellular HBsAg, HBeAg level and intracellular HBsAg, HBcAg level. The cell-penetrating bispecific antibody provides a novel approach to suppress HBV replication and secretion, which is a promising anti-HBV therapeutic antibody candidate.

## 2 Materials and methods

### 2.1 Expression and purification of recombinant HBcAg and preS1

A coding sequence corresponding to the truncated Aa1-149 HBcAg (GenBank: ACH96059.1) was constructed into the plasmid pET25b. The *Escherichia coli* strain BL21(DE3) (AlpaLifeBio, Inc.; cat. no. KTSM104L) was used for HBcAg expression. The BL21 (DE3) strain transformed with the plasmid was cultured overnight and the bacterial culture was diluted to 200 mL LB medium (containing 100 mg/l Ampicillin) at 1:100 ratio and cultured at 37 °C under shaking at 210 rpm until grown up to A600=0.8. The fresh culture was induced at 28 °C for 3 h after adding 0.2 mmol/L isopropyl-beta-d-thiogalactopyranoside (IPTG). Cells harboring HBcAg were harvested through centrifugation and crushed through ultrasonication in lysis buffer (0.2% v/v Triton X-100, 5 mM EDTA,100 mM NaCl, 50 mM Tris, pH 8.0).

Since HBcAg could self-assemble into icosahedral particles which mainly exist in inclusion bodies, the supernatant of cell lysis was discarded after centrifugation. The cell pellet containing the core particles was washed in 30 mL of lysis buffer and collected by centrifugation at 15,000 x g for 30 min at 4 °C. Subsequently, the pellet was denatured in 40 mL of dissociation buffer (50 mM sodium carbonate, 4 M urea, 10 mM 2-mercaptoethanol, 200 mM NaCl, pH 9.5) through overnight incubation at 4 °C. The supernatant containing HBcAg was collected via centrifugation and dialyzed with PBS buffer to remove the urea at 4 °C overnight.

After urea was removed, the HBcAg could self-assemble into icosahedral capsids. HBcAg capsids could be precipitated through centrifugation at 15,500 x g for 30 min at 4 °C after NH_4_SO_4_ was added at a final saturated concentration. Then the pellet was purified and HBcAg capsids were dialyzed with PBS buffer at 4 °C overnight to obtain the final HBcAg (Suffian et al., 2017).

The gene of preS1 (GenBank: AGP08994.1, Aa 1-119) was also constructed in the plasmid pET25b fused with a polyhistidine tag in the N terminal. BL21(DE3) strain was used for expression with similar culture and induction conditions. Cells were harvested and lysed via ultrasonication. After centrifugation at 15,000 x g for 30 min at 4 °C, the preS1 was purified from the supernatant using nickel-nitrilotriacetic acid (Ni-NTA) resin as recommended by the manufacturer (Qiagen Gmbh; cat. no. 31014). The protein was eluted by 50–1000 imidazole respectively (Rhyum et al., 1994).

### 2.2 Antibodies selection and recombinant expression

Two alpacas were immunized with HBcAg through subcutaneous injection once every 7 days for a total of four times. The peripheral blood from these immunized alpacas was collected and RNA within B lymphocytes cells were extracted and reverse-transcribed into cDNA. Sequences of variable domain of heavy chain of heavy-chain antibody (VHH) were amplified for phage library construction. Biopanning of nanobodies (Nabs) against HBcAg was performed three times and the positive clones from the libraries were picked up for sequencing (Yang et al., 2023). The sequences of NAbs against HBcAg were constructed into plasmids and transformed into *E. coli* strain BL21 for expression. Ni-NTA affinity columns (Qiagen Gmbh; cat. no. 31014) were used for NAbs purification and the purified NAbs were confirmed through SDS-PAGE.

For the preS1 antibody, four Balb/c mice were immunized twice with preS1. The second immunization was performed 14 days after the first one. Single B cell technologies were used for monoclonal antibody discovery. PreS1 positive Single B cell was selected by FACS and then the DNA sequence of the antibodies was gained through single-cell sequencing technologies. The antibodies variable region sequences were constructed into pcDNA3.1 vector for expression in HEK-293F cells. Mice were anaesthetized with 3% sodium pentobarbital (30 mg/kg of body weight) through intraperitoneal (IP) injection. Mice were sacrificed via cervical dislocation after the mice were intraperitoneal injected with sodium pentobarbital. All procedures were performed according to the Health Guide for Laboratory Animals (Li et al., 2023; Li et al., 2022). Ethical approval was obtained for the present study(Approval number: Shenzhen Third Hospital Lunshen Animal Zi [2024-067-01]).

### 2.3 Analysis of the reactivity of antibodies

The reactivity of antibodies was determined through chemiluminescence immunoassay. The wells were coated with 100 ng/well recombinant HBcAg or preS1 antigens. Nonspecific binding was blocked with a blocking buffer. A series of antibodies with 10-fold dilutions ranging from 0.1 to 1000 ng/mL were prepared. A total of 100μL of antibodies diluent was added to each well for 1 h incubation at 37 °C. After washing and reaction with horseradish peroxidase (HRP)-conjugated secondary antibody, chemiluminescent substrate was added and the relative light unit of luminescence (RLU) was evaluated using an Orion II Microplate Luminometer (Titertek Berthold). The following reagents were used in the chemiluminescence immunoassay: Blocking buffer (PBS with 10% sucrose and 2% bovine serum albumin); anti-mouse pAb (Thermo Fisher Scientific, Inc.; cat. no. A16066) for Anti-preS1 Fc; HRP-conjugated anti-VHH pAb (GenScript; cat. no. A01861) for Anti-HBcAg VHH; and superSignal ELISA Pico Chemiluminescent Substrate (Thermo Fisher Scientific, Inc.; cat. no. 37070). The analyses of extracellular and intracellular HBV HBsAg, HBcAg and HBeAg were performed using commercial kits (Elabscience Biotechnology, Inc.; cat. no. E-EL-H6080) (Zhang et al., 2016; Pan et al., 2016).

### 2.4 Construction of bispecific antibodies

For the construction of Anti-preS1 × Anti-HBcAg, the sequence of Anti-HBcAg VHH was fused in the C-terminus of the Anti-preS1 heavy chain and co-expressed with the light chain of Anti-preS1 in HEK-293F cells. The supernatant was collected after 5 days of transfection and purified with proteinA resin (GenScript; cat. no. L00210). For the construction of Anti-preS1 × Anti-HBcAg-R9TAT, the sequence of R9TAT (GRRRRRRRRRPPQ) was fused in the C-terminus of the Anti-preS1 × Anti-HBcAg heavy chain and co-expressed with the light chain of Anti-preS1 in HEK-293F cells. The supernatant was collected after 5 days of transfection and purified with protein A resin (GenScript; cat. no. L00210).

### 2.5 Cell culture and administration with antibodies

The HepG2.2.15 derived from hepatoblastoma HepG2 cell line with stable integration of HBV genome of the D-genotype. Dulbecco's modified Eagle's medium was used for cell culture with 10% fetal bovine serum and penicillin and streptomycin supplement (Thermo Fisher Scientific, Inc.; cat. nos. 11965092, A5670701, 15140122 and 15070063). Cells were cultured at 37 °C with 5% CO_2_ and seeded in 24 or 96-well plates at a density of 2*10^5^ cells/mL. After 24 h, the media was replaced with fresh media containing different concentrations of the antibodies respectively for evaluation of therapeutic efficacy. Quantitative analyses of HBV HBsAg, HBcAg and HBeAg level were performed using commercial kits (Elabscience Biotechnology, Inc.; cat. no. E-EL-H6080).

### 2.6 Cells and virus production

The HepAD38 cell line is an inducible human hepatoblastoma cell line harbouring an integrated 1.2-fold tetracyclineresponsive HBV genome (serotype ayw, genotype D)(25). For the production of HBV particles, HepAD38 cells were grown in William's E medium supplemented with 2% dimethyl sulfoxide (DMSO), Upon removal of tetracycline from the culture medium, the cells secrete virus into the supernatant, the supernatant was filtered by a 0.45μm filter, 30mL of supernatant was add to centrifuge tube, 5mL of 20% sucrose solution was slowly inject into the bottom of the centrifuge tube with a syringe, centrifuge at 100,000 g 4 °C for 16 hours, the centrifuged HBV was re-suspended with 200 μL OPTI-MEM medium (Gibco/Invitrogen) aliquoted and stored at -80 °C for further experiments. HepG2-NTCP cells were maintained in Dulbecco's modified Eagle medium (DMEM; Gibco/Invitrogen) supplemented with 10% fetal bovine serum (FBS) (Gibco/Invitrogen) and 5 μg/mL puromycin (Thermo Fisher Scientific), at 37C, under an atmosphere containing 5% CO2.

### 2.7 HBV infection test

HepG2-NTCP cells were seeded in 96 well plant at 4 × 10^4^ cells/well, cultured in a 5% CO2 incubator at 37 °C for 24h. 5 × 10^7^ copies of genome equivalent HBV which produced from HepAD38 cell line (serotype ayw, genotype D), was added to each well with 50 μg/mL Anti-HBcAg Fc,Anti-HBcAg Fc-R9TAT,Anti-preS1 × Anti-HBcAg ,Anti-preS1 × Anti-HBcAg-R9TAT and control antibody respectively, the supernatant was removed after 24 hours administration, washed three times with cold PBS buffer, and then added 100 μL William's E medium supplemented with 2.5% dimethyl sulfoxide (DMSO) supplemented with 20% fetal bovine serum (FBS). 24 hours later, the supernatant was removed and washed three times with cold PBS buffer, and then added 100 μL William's E medium supplemented with 2.5% DMSO supplemented with 20% FBS for six days. In the sixth days, the supernatant was collected for detection. Quantitative analyses of HBV HBsAg and HBeAg level were performed using commercial kits (Elabscience Biotechnology, E-EL-H6080; Lianzu Biology, LZ-E028718). The detection of HBsAg and HBeAg level ‌ is based on ‌ double antibody sandwich method ‌ and chemiluminescence enzyme immunoassay (CLEIA). Briefly, the antigen specific antibodies (capture antibodies) were coated in the plate, which then bind to the corresponding antigens in the sample to form an immune complex. After washing, HRP-labeled antibodies (detection antibodies) are added to bind to the antigens in the immune complex to form an HRP-labeled antibody-antigen-solid phase antibody complex. Finally, add the substrate to evaluate the relative light unit (RLU) using an Orion II Microplate Luminometer (Berthold, Germany) following the addition of SuperSignal ELISA Pico Chemiluminescent Substrate (Thermo Scientific, Rockford, USA). The antigen standard provided in the kit is used for quantitative testing.

### 2.8 Statistical analysis

GraphPad Prism 7.0 (Dotmatics) was used for statistical analysis. The unpaired *t*-test was used for continuous variables comparison. Differences were considered significant at a two-sided P<0.05.

## 3 Results

### 3.1 Expression and purification of recombinant truncated HBcAg and PreS1

**Fig. 1:**
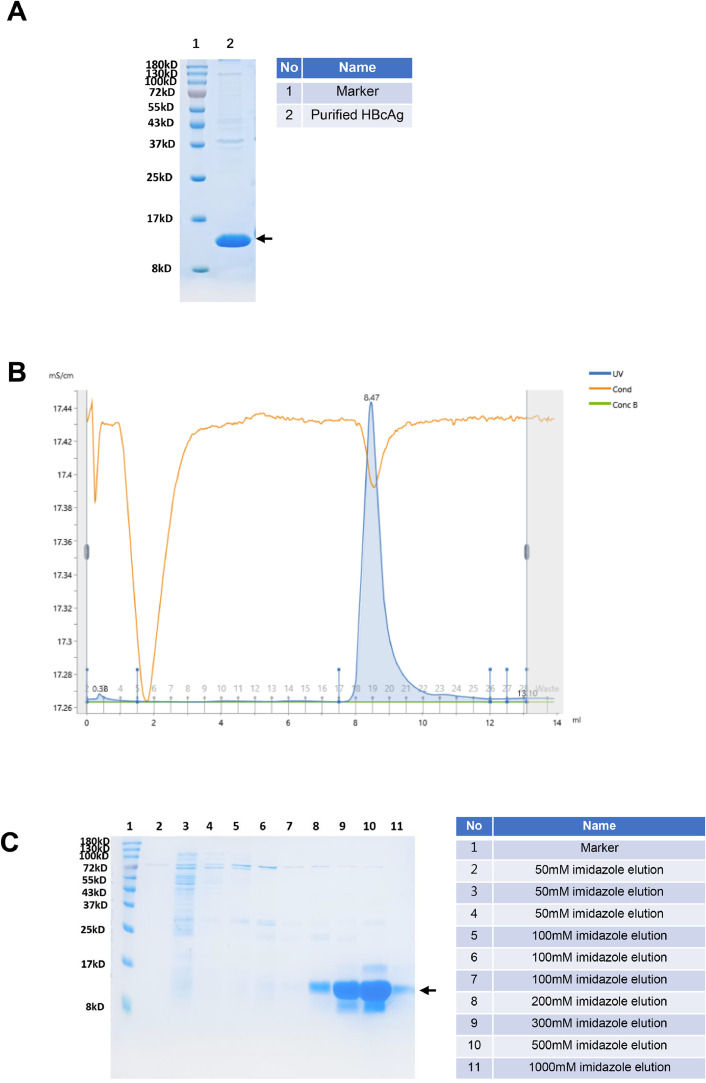
Expression and purification of HBcAg and preS1.

The coding sequence of HBcAg (GenBank: ACH96059.1, Aa 1-149) was constructed into the vector pET25b and the expression was performed in BL21 *E. coli* strain (DE3). Purified HBcAg(Aa 1-149) was stained by coomassie bright blue after SDS-PAGE (Fig. 1A) and analyzed through Size-Exclusion Chromatography (SEC) with superdex 200 increase 10/300 GL (Fig. 1B). The peak value was at 8.47 mL, revealing the molecular weight is above 669 KDa, which suggested dissociated HBcAg(Aa 1-149) could reassemble into particles. These particles were used for the immunization study.

A, The purified HBcAg was analyzed by 12% SDS-PAGE with coomassie blue-stain, the molecular weight is 16.88 kDa; B, the purified HBcAg was analyzed by SEC with superdex 200 increase 10/300 GL, it shows that the peak value is at 8.47mL, which indicated that the total molecular weight is above 669 KDa, revealed that HBcAg re-associate into particles; C, the preS1 purified by affinity chromatography with Ni resin were eluted by 50–1000 imidazole respectively, the molecular weight is 13.38 kDa. The target protein was marked with arrow.

The coding sequence of preS1 (GenBank: AGP08994.1, Aa 1-119) was constructed into the plasmid pET25b fused with a polyhistidine tag in the N terminal. The *E. coli* strain BL21(DE3) was also used for preS1 Expression. The preS1 protein was purified using Ni-NTA resin from the supernatant of cell lysis and eluted with 50–1000 mM imidazole respectively (Fig. 1C). The purity of the HBcAg and preS1 were >90%, as indicated by the Coomassie bright blue stain performed after SDS-PAGE. The concentration of the recombined proteins was determined using the Bradford assay (Bradford,1976).

### 3.2 Anti-HBcAg and Anti-preS1 selection, expression and reactivity

**Fig. 2:**
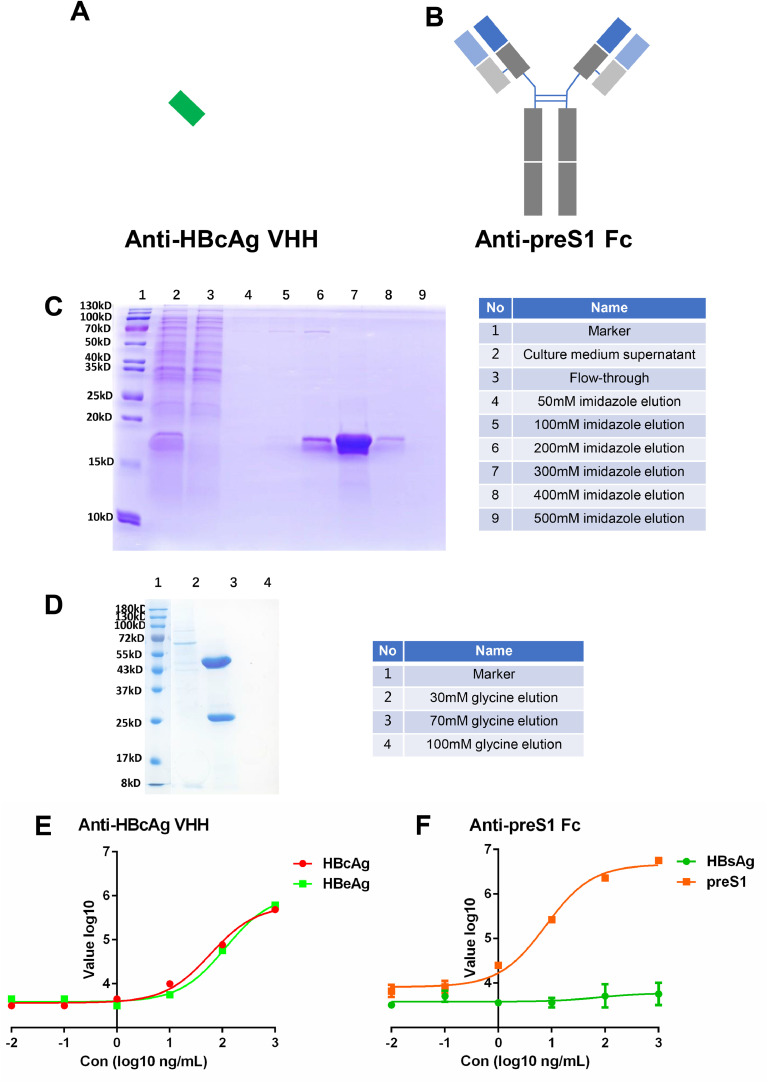
Anti-HBcAg and Anti-preS1 selection,expression and reactivity.

Alpacas were immunized with HBcAg. Phage display technology was used to select NAbs against HBcAg. The Monoclonal phage ELISA of HBcAg was shown in Supplement Table 1. Finally, the one with the highest affinity from 12 Anti-HBcAg VHHs was chosen for subsequent research. The VHH form of Anti-HBcAg without the Fc region was used in the present study (Fig. 2A). The VHHs were expressed in *E. coli* and Ni-NTA affinity columns were used for purification. The purified NAbs were confirmed through SDS-PAGE with coomassie blue stain (Fig. 2C).

A, the diagram of Anti-HBcAg VHH; B, the diagram of Anti-preS1 Fc; C, Anti-HBcAg VHH was expressed in E.coli and purified with Ni resin, the elution were analyzed by 15% SDS-PAGE with coomassie blue-stain, the molecular weight is 13.42 kDa; D, Anti-preS1 Fc was expressed in HEK-293F cells and purified with proteinA resin, the elution were analyzed by 10% SDS-PAGE with coomassie blue-stain, the molecular weight is 146.64 kDa; E, the reactivity of Anti-HBcAg VHH with HBcAg and HBeAg was tested by ELISA; F, the reactivity of Anti-preS1 Fc with preS1 and HBsAg was tested by ELISA.

The Anti-preS1 antibodies were selected from mice through single B cell technologies. The DNA sequence of the antibodies was obtained through single-cell sequencing of positive single B cells. The antibody variable region sequences were constructed into pcDNA3.1 vector with Fc region (Fig. 2B). Antibodies were expressed in HEK-293F cells (Fig. 2D). A total of eight Anti-preS1 candidates were selected and the clone with the highest affinity for was chosen for subsequent research.

ELISA demonstrated that Anti-HBcAg VHH could react with both HBcAg and HBeAg (Fig. 2E). Anti-preS1 Fc antibody could react only with preS1 but not HBsAg (Fig. 2F).

### 3.3 Construction of cell-penetrating Anti-HBcAg antibody

**Fig. 3:**
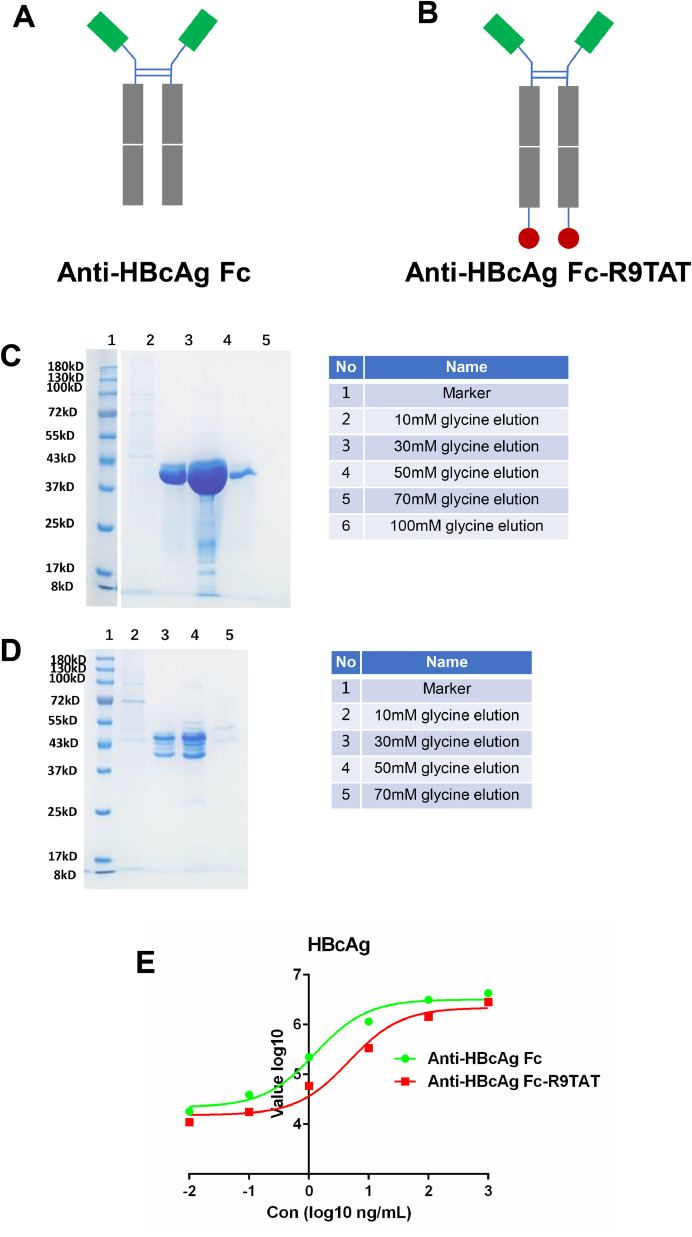
Anti-HBcAg Fc and Anti-HBcAg Fc-R9TAT expression and reactivity.

The native antibody could hardly internalize into live cells. To enhance its cell-penetrating ability, the Anti-HBcAg Fc heavy chain was fused with the CPP R9TAT (GRRRRRRRRRPPQ) on the C-terminus and named Anti-HBcAg Fc-R9TAT (Fig. 3A and B). Both Anti-HBcAg Fc and Anti-HBcAg Fc-R9TAT were constructed into pcDNA3.1 vector and expressed in HEK-293F cells (Fig. 3C and D). The band of Anti-HBcAg Fc-R9TAT is dispersed around 43 KDa, possibly due to different glycosylation level. The reactivity of Anti-HBcAg Fc and Anti-HBcAg Fc-R9TAT with HBcAg was tested using ELISA, as shown in Fig. 3E. Both Anti-HBcAg Fc and Anti-HBcAg Fc-R9TAT could react with HBcAg, while Anti-HBcAg Fc showed an improved performance.

A, the diagram of Anti-HBcAg Fc; B, the diagram of Anti-HBcAg Fc-R9TAT; C, Anti-HBcAg Fc was expressed in HEK-293F cells and purified with proteinA resin, the elution were analyzed by 10% SDS-PAGE with coomassie blue-stain, the molecular weight is 77.74 kDa; D, Anti-HBcAg Fc-R9TAT was expressed in HEK-293F cells and purified with proteinA resin, the elution were analyzed by 10% SDS-PAGE with coomassie blue-stain, the molecular weight is 81.32 kDa; E, the reactivity of Anti-HBcAg Fc and Anti-HBcAg Fc-R9TAT with HBcAg was detected by ELISA.

### 3.4 Construction of cell-penetrating bispecific antibody to improve specificity

**Fig. 4:**
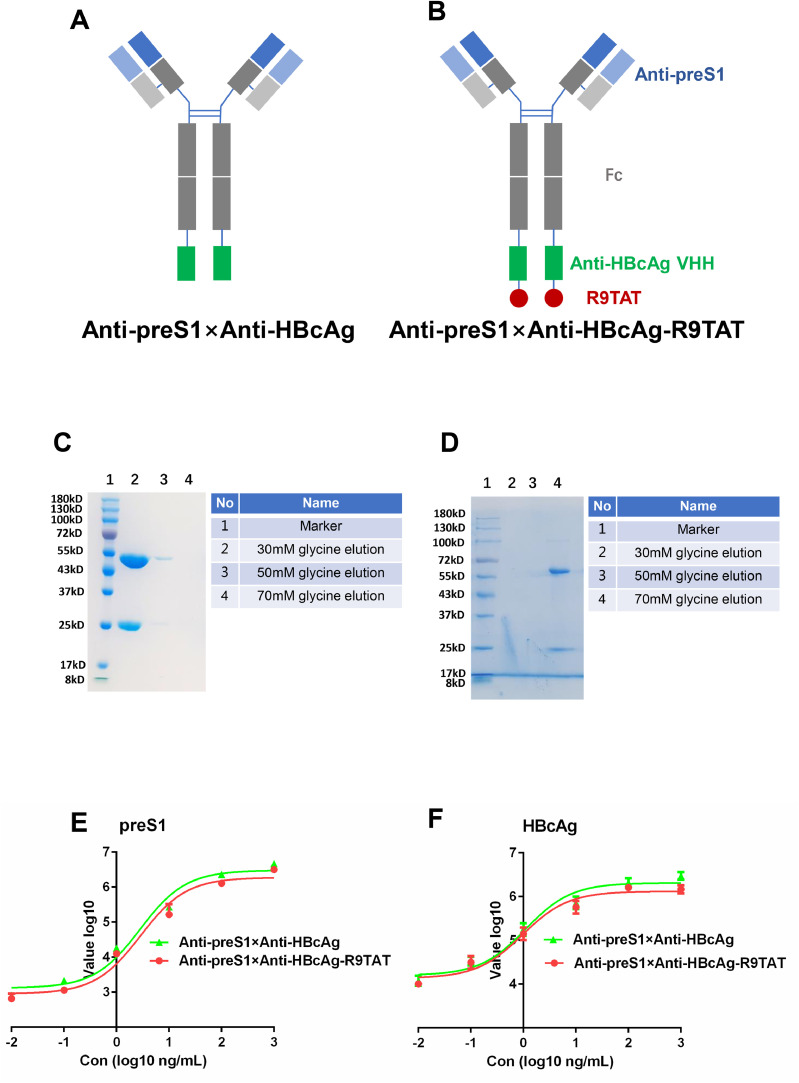
Cell-penetrating bispecific antibody construction, expression and reactivity.

CPPs could deliver antibodies into the cell but have no cell specificity. To enhance the internalization of the Anti-HBcAg Fc-R9TAT in HBV-infected hepatocytes, the present authors constructed the Anti-preS1 × Anti-HBcAg bispecific and Anti-preS1 × Anti-HBcAg-R9TAT bispecific antibodies (Fig. 4A and B). The Anti-HBcAg VHH was further fused in the C-terminus of the Anti-preS1 Fc heavy chain to obtain a new antibody named Anti-preS1 × Anti-HBcAg. R9TAT was fused in the C-terminus of Anti-preS1 × Anti-HBcAg, to obtain a new antibody named Anti-preS1 × Anti-HBcAg-R9TAT. Anti-preS1 × Anti-HBcAg and Anti-preS1 × Anti-HBcAg-R9TAT were constructed into pcDNA3.1 vector with Fc region respectively, then expressed in HEK-293F cells (Fig. 4C and D). The reactivity of Anti-preS1 × Anti-HBcAg and Anti-preS1 × Anti-HBcAg- R9TAT with preS1 and HBcAg were tested using ELISA (Fig. 4E and F). Both Anti-preS1 × Anti-HBcAg and Anti-preS1 × Anti-HBcAg- R9TAT could react with preS1 and HBcAg.

A, the diagram of Anti-preS1 × Anti-HBcAg, the Anti-HBcAg VHH was fused in the C-terminus of the heavy chain of Anti-preS1 Fc; B, the diagram of Anti-preS1 × Anti-HBcAg-R9TAT, R9TAT was fused in the C-terminus of the heavy chain of Anti-preS1 × Anti-HBcAg; C, Anti-preS1 × Anti-HBcAg was expressed in HEK-293F cells and purified with proteinA resin, the elution were analyzed by 10% SDS-PAGE with coomassie blue-stain, the molecular weight is 174.56 kDa; D, Anti-preS1 × Anti-HBcAg-R9TAT was expressed in HEK-293F cells and purified with proteinA resin, the elution were analyzed by 10% SDS-PAGE with coomassie blue-stain, the molecular weight is 183.78 kDa; E, the reactivity of Anti-preS1 × Anti-HBcAg and Anti-preS1 × Anti-HBcAg- R9TAT with preS1 were tested by ELISA;F, the reactivity of Anti-preS1 × Anti-HBcAg and Anti-preS1 × Anti-HBcAg- R9TAT with HBcAg were tested by ELISA.

### 3.5 Cell-penetrating and cell specificity character of Anti-Pres1 × Anti-HBcAg-R9TAT

**Fig. 5:**
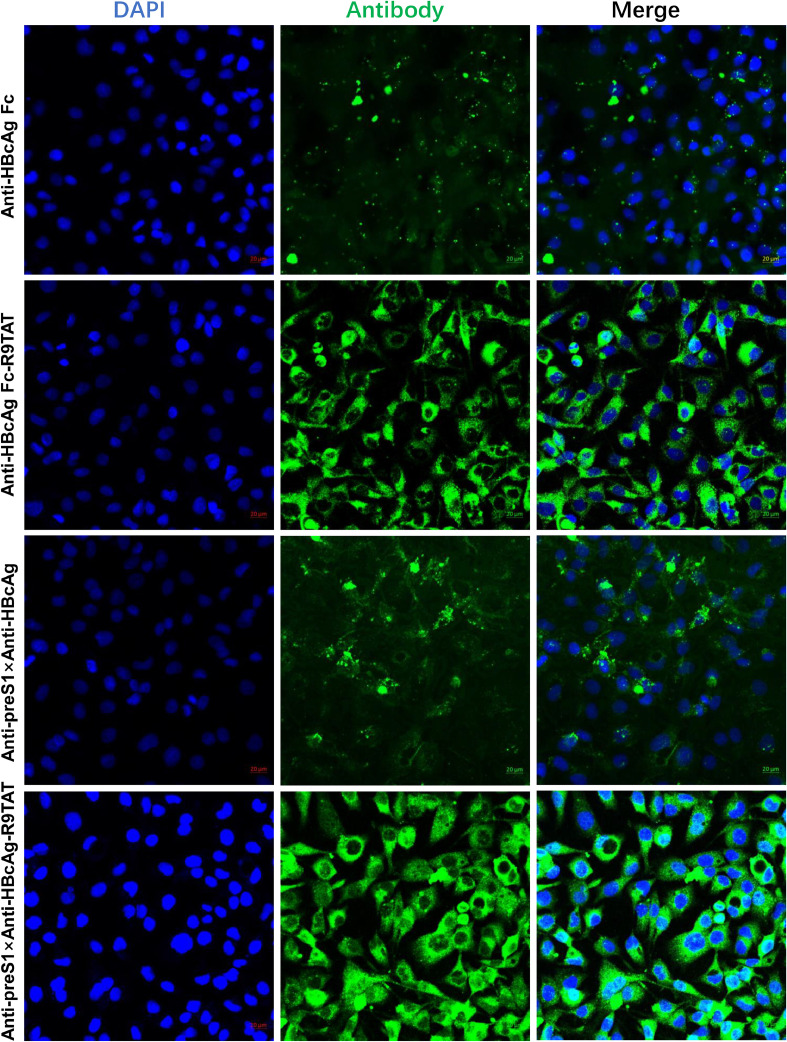
The cell-penetrating efficiency of these antidodies.

The present authors compared the cell penetration and tropism character of the four antibodies, namely Anti-HBcAg Fc, Anti-HBcAg Fc-R9TAT, Anti-preS1 × Anti-HBcAg and Anti-preS1 × Anti-HBcAg-R9TAT (Fig. 5). HepG2.2.15 cells with stable HBV expression were used. The fluorescence intensity of Anti-HBcAg Fc-R9TAT and Anti-preS1 × Anti-HBcAg-R9TAT was increased compared with that of Anti-HBcAg Fc and Anti-preS1 × Anti-HBcAg, respectively, revealing that R9TAT could improve the antibody internalization. The fluorescence of Anti-preS1 × Anti-HBcAg-R9TAT was brighter than that of Anti-HBcAg Fc-R9TAT, revealing that bispecific antibody could improve the antibody tropism.

We used HepG2.2.15 to compare the cell-penetrating of the four antibodies: Anti-HBcAg Fc,Anti-HBcAg Fc-R9TAT,Anti-preS1 × Anti-HBcAg and Anti-preS1 × Anti-HBcAg-R9TAT. We treated HepG2.2.15 with 10μg/mL of the four antibodie for 12 hours respectively, then the cells were washed three times and fixed for immunofluorescence assay. Anti-mouse IgG-FITC was used as detection antibody, DAPI strain for nucleus. We used confocal microscope (ZEISS)to test the immunofluorescence.

**Fig. 6:**
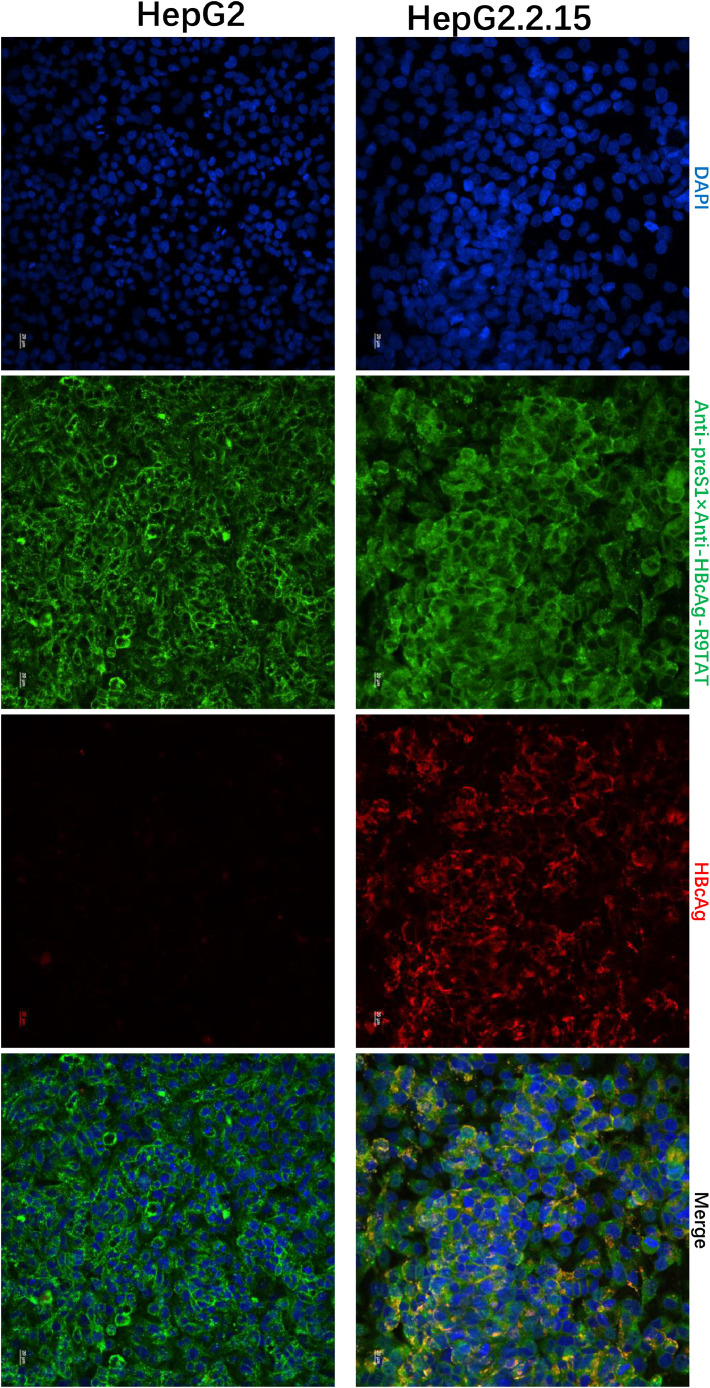
Cell specificity character.

We perform a quantitative analysis of the obtained microscopic images using Image J software, the mean fluorescence intensity was calculated in Supplement Fig. 1. To investigate whether the cell-penetrating bispecific antibodies specifically enter cells expressing the HBV virus, their entry into HEPG2.2.15 cells was compared with the parental HepG2 line, as shown in Fig. 6, the fluorescence intensity of Anti-preS1 × Anti-HBcAg-R9TAT in HepG2.2.15 cells is brighter than that of HepG2 cells, indicated that the cell-penetrating bispecific antibodies ends to enter HBV containing cells. We also found that Anti-preS1 × Anti-HBcAg-R9TAT could internalize into the HepG2.2.15 and colocalize with HBcAg, indicated that the Anti-preS1 × Anti-HBcAg-R9TAT could bind the inner HBcAg (Supplement Fig. 2).

To investigate whether the cell-penetrating bispecific antibodies specifically enter cells expressing the HBV virus, their entry into HEPG2.2.15 cells was compared with the parental HepG2 line, the HepG2.2.15 derived from hepatoblastoma HepG2 cell line with stable integration of HBV genome. Immunofluorescence confocal laser scanning microscopy to assess the intracellular localizations of internalized Anti-preS1 × Anti-HBcAg-R9TAT and HBcAg. We treated HepG2.2.15 and HepG2 cells with 10μg/mL of Anti-preS1 × Anti-HBcAg-R9TAT for 12 hours respectively, then the cells were washed three times and fixed for immunofluorescence assay. Anti-mouse IgG-FITC was used to detect Anti-preS1 × Anti-HBcAg-R9TAT, Anti-HBcAg rabbit polyclonal antibodies and Anti-rabbit-AF647 secondary antibodies was used to detect the inner HBcAg, DAPI strain for nucleus.

### 3.6 Therapeutic effect of the cell-penetrating bispecific antibody in HepG2.2.15

**Fig. 7:**
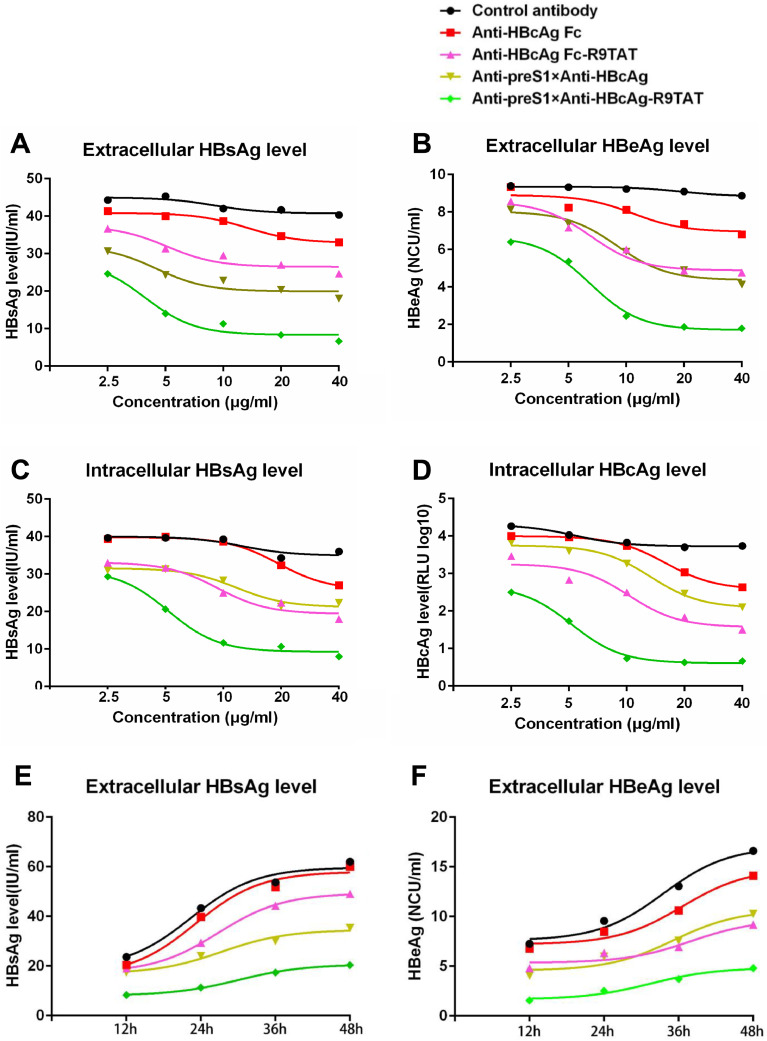
Therapeutic effect of the cell-penetrating bispecific antibody in HepG2.2.15.

To investigate the therapeutic effect of these antibodies against HBV, we first tested in the HBV genome integrated stable cells HepG2.2.15, which constitutively produces HBV virus. HepG2.2.15 cells were treated with different concentration (2.5,5,10,20 or 40 μg/mL) of five antibodies, namely Anti-HBcAg Fc, Anti-HBcAg Fc-R9TAT, Anti-preS1 × Anti-HBcAg, Anti-preS1 × Anti-HBcAg-R9TAT and control antibody, for 24 h respectively. The control antibody was Anti-SARS-CoV-2 RBD antibody (IBT-CoV144), which was reported in our former study (Yang et al., 2023). The culture supernatant was collected and tested for HBsAg and HBeAg level, we found that the cell-penetrating bispecific antibody (Anti-preS1 × Anti-HBcAg-R9TAT) could suppress the extracellular HBsAg and HBeAg level significantly compared with the other antibodies, it was concentration-dependent (Fig. 7A and B). Meanwhile, Anti-preS1 × Anti-HBcAg-R9TAT could reduce both the intracellular HBsAg and HBcAg level significantly compared with the other antibodies (Fig. 7C and D). We added the detection of HBsAg and HBeAg in culture supernatant at sequential time intervals, 10 μg/mL of the five antibodies treated HepG2.2.15 cells, the culture supernatant were collected at 12, 24, 36 and 48 hours respectively. Not only the extracellular HBsAg but also the HBeAg were remarkable reduced (Fig. 7E and F). For statistical analysis, we selected the data from HepG2.2.15 cells treated with 10 μg/mL of the five antibodies for 24 hours, the unpaired *t*-test was used for continuous variables comparison, it showed that the cell-penetrating bispecific antibody could suppess not only the extracellular HBsAg and HBeAg level but also the intracellular HBsAg and HBcAg level significantly (supplementary Figure 3).

HepG2.2.15 was treated with different concentration (2.5,5,10,20 or 40 μg/mL) of the five antibodies: Anti-HBcAg Fc,Anti-HBcAg Fc-R9TAT,Anti-preS1 × Anti-HBcAg,Anti-preS1 × Anti-HBcAg-R9TAT and control antibody, for 24 h respectively. The culture supernatant was collected and tested for extracellular HBsAg(A) and extracellular HBeAg(B) level by ELISA. The cell precipitation was collected and lysis to detect the intracellular HBsAg(C) and HBcAg level(D). We added the detection of HBsAg and HBeAg in culture supernatant at sequential time intervals, 10 μg/mL of the five antibodies treated HepG2.2.15 cells, the culture supernatant were collected at 12, 24, 36 and 48 hours respectively. The culture supernatant was collected and tested for extracellular HBsAg(E) and extracellular HBeAg(F) level by ELISA.

### 3.7 Therapeutic effect of the cell-penetrating bispecific antibody in HBV infection model

**Fig. 8:**
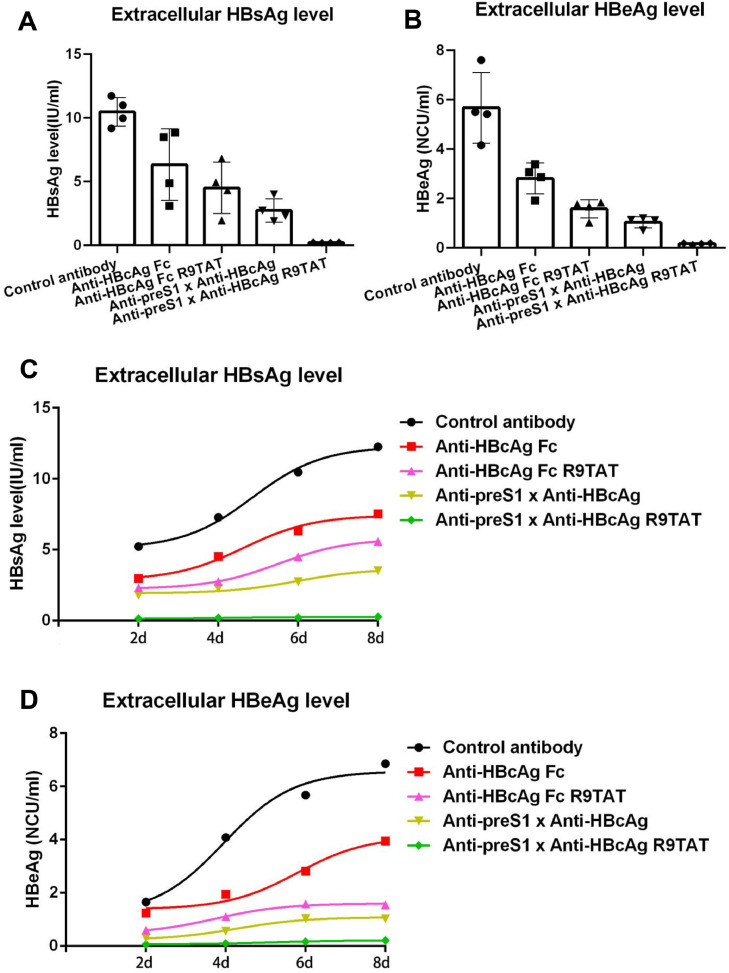
Therapeutic effect in HBV infection model.

To investigate the therapeutic effect in HBV infecting HepG2-NTCP cells model, we produced HBV virus through the HBV-inducible cell line HepAD38, the virus was preparated from the culture supernatant by sucrose density gradient centrifugation. The HepG2-NTCP cells were infected with virus treated with 50 μg/mL Anti-HBcAg Fc,Anti-HBcAg Fc-R9TAT,Anti-preS1 × Anti-HBcAg ,Anti-preS1 × Anti-HBcAg-R9TAT and control antibody respectively. As shown in Fig. 8A and B, the cell-penetrating bispecific antibody could significantly suppress the extracellular HBsAg and HBeAg level than the other antibodies. We also detected the HBsAg and HBeAg level in culture supernatant at sequential time intervals, 50 μg/mL of the five antibodies treated HepAD38 cells after HBV infection, the culture supernatant was collected at 2, 4, 6 and 8 days respectively. Not only the extracellular HBsAg but also the HBeAg were remarkable reduced (Fig. 8C and D). These results demonstrated that the cell-penetrating bispecific antibody could suppress HBV replication and secretion, revealing a new promising anti-HBV therapeutic antibody candidate.

We investigated the therapeutic effect of these antibodies in HBV infection model, the HepG2-NTCP cells were infected with HBV virus treated with 50 μg/mL Anti-HBcAg Fc,Anti-HBcAg Fc-R9TAT,Anti-preS1 × Anti-HBcAg ,Anti-preS1 × Anti-HBcAg-R9TAT and control antibody respectively. The culture supernatant was collected and tested for extracellular HBsAg(A) and HBeAg(B) level by ELISA. P values were calculated using a two-sided unpaired t test, * indicates p<0.05,** indicates p<0.01 and ns indicates p>0.05. We also detected the HBsAg and HBeAg level in culture supernatant at sequential time intervals, 50 μg/mL of the five antibodies treated HepAD38 cells after HBV infection, the culture supernatant was collected at 2, 4, 6 and 8 days respectively.

## 4 Discussion

HBcAg composes the nucleocapsid of HBV and the reverse transcription of pregenome RNA and second-strand synthesis are known to take place in the nucleocapsid which are essential steps for viral replication. The CAMs can disturb pgRNA encapsidation and subsequent replication by misdirecting capsid-like structure formation (Peters and Locarnini, 2017). The aforementioned CAMs are small molecules, while the therapeutic antibodies are a different type of CAMs. It has been reported that intracellular transfection of anti-HBc ScFv plasmid could inhibit viral replication in the human hepatoblastoma-derived cell line HB611 which produces HBV and both single-stranded and partially double-stranded DNA intermediates were significantly suppressed in the cells, suggesting that intracellular anti-HBc ScFv could inhibit HBV DNA replication, this was the proof of concept study for targeting the intracellular HBcAg by antibodies (Wang et al., 2015). Blocking the HBV nucleocapsid assembly is an attractive strategy for novel anti-HBV agents, however, it is limited by suitable delivery vector. The major challenge faced with intrabody expression through the use of recombinant DNA technology is the safety concerns associated with its application in human clinical therapy. The therapeutic regulation of intracellular targets is largely based on small molecules because the cell membranes are impermeable to most macromolecules. Antibodies have high-binding specificity and minimal off-target effects, but they can hardly penetrate living cells naturally (Weisbart et al., 2012). How to improve the efficiency of antibody entering cells is an important research objective of our study. CPPs are a powerful tool to deliver therapeutic macromolecules across the cell membranes, Xun *et al* reported that intracellular delivery of anti-HBc scFv fused with CPP, called transbody, inhibited HBV replication in HepG2.2.15 cells, which could interfere with the assembly of nucleocapsid and significantly reduced both the supernatant and the intracellular DNA level (Xun et al., 2013; Li et al., 2017).

In our study, we selected R9TAT, a membrane penetrating peptide with higher membrane penetration efficiency, revealing that R9TAT could remarkable improve the antibody internalization (Fig. 5).

CPP has no cell specificity, transbody fused with CPP could enter into different cell types, which may cause off-target and cytotoxicity. A cell-penetrating bispecific antibody was designed to overcome this concern: with one antibody moiety binding to the surface target and the other binding to the intracellular target along with CPP fusion. The constructed cell-penetrating bispecific antibody could penetrate the target cells and recognize the inner protein. Weisbart et al. (2012) reported a cell-penetrating bispecific antibody consisting of a cell-penetrating anti-DNA autoantibody with Anti-MDM2 mAb. They showed that the bispecific antibody retains cell-penetrating and MDM2-binding activity, it could increase inner p53 level and inhibit MDM2-addicted tumors1 growth. However, this reported cell-penetrating bispecific antibody binds to DNA and MDM2, both of which are inner targets, whilst in the present study preS1 was selected as the surface target and HBcAg as the inner target. These antibodies were fused with R9TAT to create the Anti-preS1 × Anti-HBcAg-R9TAT, a new form of cell-penetrating bispecific antibody that could not only recognize surface preS1 but also inner HBcAg. We found that the fluorescence of Anti-preS1 × Anti-HBcAg-R9TAT was brighter than that of Anti-HBcAg Fc-R9TAT in HepG2.2.15 cells, revealing that bispecific antibody could improve the antibody tropism (Fig. 5).

We utilised two system, HBV stable cell line HepG2.2.15 and HBV infecting HepG2-NTCP model to investigate the therapeutic effect of these antibodies. The results obtained by these two systems revealing that the cell-penetrating bispecific antibody could suppress not only the extracellular HBsAg and HBeAg but also the intracellular HBsAg and HBcAg level significantly (Fig. 7, Fig. 8). It is unclear why the anti-HBcAg transbody could decrease HBV DNA and viral proteins, maybe the antibody could block the nucleocapsid assembly and HBV DNA transcription, which are major steps for virus replication. In the process of nucleocapsid assembly, HBcAg could encapsidate the pgRNA, host factors and reverse transcriptase to form an icosahedral nucleocapsid, which provides pgRNA reverse transcription into viral DNA. The possible mechanism of the anti-HBcAg transbody is that once enters the cells the antibody binds to HBcAg so that the pgRNA cannot package and reverse transcribed into DNA, thereby suppressing virus replication.

The majority of reported cell-penetrating antibodies are in ScFv or VHH form from which Fc is removed to reduce antibody size and increase the cell-penetrating efficiency. The Fc regain of antibodies is crucial for Fc-dependent effector function. The infected cells bound by antibodies can be eliminated by innate immune cells through three types of mechanisms: Antibody-dependent cellular phagocytosis; antibody-dependent cellular cytotoxicity; or complement-dependent cytotoxicity. These Fc-dependent effector functions of HBV antibodies would contribute to eliminating infected hepatocytes, thereby diminishing *de novo* production of virions. At the same time, the intracellular immunity of inner pathogens clearance is very important. The cytosolic Fc receptor Tripartite motif containing-21 (TRIM21) is essential to initiate clearance of intracellular antibody-bound invading non-enveloped viruses. Upon engagement of the Fc moiety of antibodies bound to viruses, TRIM21 triggers virus degradation by the proteasome and innate immune signaling response that prevents viral replication (Rhodes and Isenberg, 2017). In our previous study, we designed an anti-HBx cell-penetrating whole molecule antibody, which could suppress HBV via a TRIM21-dependent pathway and found that the Fc region was necessary because TRIM21 bound to the Fc of antibody (Zhang et al., 2018). The reported intrabody and transbody for HBcAg lack the Fc region, which cannot trigger the TRIM21-dependent coordinated effector or signaling response that prevents viral replication. In the present study, the Fc was added to the cell-penetrating bispecific antibody and it was found significant Anti-HBV effect. Another reason for adding Fc to the cell-penetrating bispecific antibody is that the Fc region could increase the half-life *in vivo* through binding to the Fc receptor. The cell-penetrating bispecific antibody Anti-preS1 × Anti-HBcAg-R9TAT has an Fc region of the human IgG1 subtype; this design could trigger Fc-dependent effector functions *in vivo*. The present study provided a promising approach against human chronic HBV infection and valuable novel development against intracellular targets.

## 5 Conclusion

The cell-penetrating bispecific antibody Anti-preS1 × Anti-HBcAg-R9TAT could not only recognize preS1 and HBcAg but also internalize into living cells efficiently, suppressing the extracellular HBsAg, HBeAg and intracellular HBsAg, HBcAg *in vitro*, providing a novel approach to suppress HBV replication and secretion and is a promising anti-HBV therapeutic antibody candidate.

## Ethics approval and consent to participate

Ethical approval was obtained for the present study(Approval number: Shenzhen Third Hospital Lunshen Animal Zi [2024-067-01]).

## Patient consent for publication

Not applicable.

## Funding

This work was supported by grants from the Shenzhen Science and Technology Program (JCYJ20200109144201725, JCYJ20220530163412028, KJZD20240903101359020), Shenzhen High-level Hospital Construction Fund (23250G1003), Shenzhen Medical Research Funds (B2303001), Medical Research Foundation of Guangdong Province [grant no. B2022270] and 10.13039/501100003453Natural Science Foundation of Guangdong Province [grant no. 2023A1515010253].

## CRediT authorship contribution statement

**Chongwei Xie:** Investigation. **Bing Zhou:** Methodology. **Da Yao:** Visualization, Resources. **Xin Wang:** Methodology, Investigation. **Lihong Zhong:** Resources. **Chuanghua Qiu:** Formal analysis. **Junfang Zhang:** Conceptualization.

## Declaration of competing interest

The authors declare no competing interests.

## Data Availability

The authors confirm that the data supporting the findings of this study are available within the article and/or its supplementary materials.

## Appendix Supplementary materials

Image, application 1

Image, application 2
