# Supplementary material for: A cell-penetrating bispecific antibody suppresses hepatitis B virus replication and secretion
Source: Virus Res. 2025 Jan 31;353:199531. doi: 10.1016/j.virusres.2025.199531 (PMC11841211; doi:10.1016/j.virusres.2025.199531)
Supplement: Supplementary file 1 [file mmc1.pdf]

深圳市第三人民医院科研项目实验动物福利和伦理审查批准文件

Approval Document for Welfare and Ethical Review of Experimental Animals in the Research Project of Shenzhen Third People's Hospital

批件号：深圳三院伦审动物字[2024-067-01]号

Approval number: Shenzhen Third Hospital Lunshen Animal Zi [2024-067-01]

<table>
<tr><td>项目名称
project name</td><td colspan="3">基 2020N089 靶向 HBV 双特异性抗体研究
Research on targeted HBV bispecific antibody based on 2020N089</td></tr>
<tr><td>项目来源
project source</td><td colspan="3">深圳市科技创新委员会
Shenzhen Science and Technology Innovation Committee</td></tr>
<tr><td>申请科室
Application Department</td><td>肝病研究所
Liver Disease Research Institute</td><td>申请人
applicant</td><td>张政
Zheng Zhang</td></tr>
<tr><td>审查方式
Review method</td><td>简易审查
Expedited review</td><td>审查时间
Review time</td><td>2024 年 05 月 22 日
May 22, 2024</td></tr>
<tr><td>审查类型
Review type</td><td colspan="3">初始审查
Initial review</td></tr>
<tr><td>审查内容
Review content</td><td colspan="3">1、实验动物伦理审查申请表
1. Application Form for Ethical Review of Experimental Animals
2、研究方案（版本号：1.0；版本日期：2024 年 04 月 22 日）
2. Research Protocol (Version Number: 1.0; Version Date: April 22, 2024)
3.1、科学性审查证明文件
3.1 Scientific Review Certificate Documents
3.2、立项证明 1
3.2 Project Approval Certificate 1
3.3、立项证明 2
3.3 Project Approval Certificate 2
3.4、科学性审查证明文件
3.4 Scientific Review Certificate Documents
4、研究承诺函
4. Research Commitment Letter
5、培训证书
5. Training certificate
6、授权书（版本号：1.0；版本日期：2024 年 01 月 17 日）
6. Authorization Letter (Version Number: 1.0; Version Date: January 17, 2024)
7、实验动物伦理审查申请表签字扫描版
7. Scanned version of the signed application form for ethical review of experimental animals</td></tr>
<tr><td>审查意见
Review opinions</td><td colspan="3">1、根据《实验动物管理条例》（2017 年修订版）、《关于善待实验动物指导性意见》和《实验动物 动物实验通用要求》（GB/T35823-2018）等规定和要求：经本伦理委员会审查，本项目符合实验动物伦理的要求，意见如下：</td></tr>
</table>

	1. According to the regulations and requirements of the "Regulations on the Management of Experimental Animals" (revised in 2017), "Guiding Opinions on the Treatment of Experimental Animals", and "General Requirements for Experimental Animal Experiments" (GB/T35823-2018), etc., after review by the Ethics Committee, this project meets the ethical requirements of experimental animals. The opinions are as follows:<br>■批准 approved ☐修改后批准 Approved after modification<br>☐修改后重审 Re examination after modification<br>☐不批准 Not Approved<br>☐终止或暂停已经开展的动物实验 Terminate or suspend animal experiments that have already been conducted<br>2、《伦理审批批准文件》是唯一的项目通过伦理审查的证明文件；<br>2. The 'Ethical Approval Document' is the only proof document that the project has passed the ethical review;<br>3、《伦理审批批准文件》原件一式两份，研究者一份，伦理委员会一份；<br>3. Two original copies of the 'Ethical Approval Document', one for the researcher and one for the ethics committee;	
	**年度定期/跟踪审查频率**<br>**Annual Regular/Tracking Review Frequency**	12 个月<br>12 months
	**批件有效期**<br>**Validity period of approval documents**	2024-05-22~2025-05-21
	主任委员（签名）：张国良<br>Chairman (signature): Guoliang Zhang<br>深圳市第三人民医院实验动物伦理委员会<br>Experimental Animal Ethics Committee of Shenzhen Third People's Hospital<br>2024 年 05 月 22 日<br>May 22, 2024	

联系方式：
contact information:
地址：深圳市龙岗区布吉镇布澜路 29 号
Address: No. 29 Bulan Road, Buji Town, Longgang District, Shenzhen
邮编：518112
Postal Code: 518112
电话：18820242267
Phone: 18820242267

提示：请研究者持《伦理审批批准文件》到科教部完成备案

Reminder: Researchers are required to bring the "Ethical Approval Document" to the Ministry of Science and Education for filing

深圳市第三人民医院科研项目实验动物福利和伦理审查批准文件

批件号：深圳三院伦审动物字[2024-067-01]号

<table>
<tr><td>项目名称</td><td colspan="3">基2020N089 靶向HBV双特异性抗体研究</td></tr>
<tr><td>项目来源</td><td colspan="3">深圳市科技创新委员会</td></tr>
<tr><td>申请科室</td><td>肝病研究所</td><td>申请人</td><td>张政</td></tr>
<tr><td>审查方式</td><td>简易审查</td><td>审查时间</td><td>2024年05月22日</td></tr>
<tr><td>审查类型</td><td colspan="3">初始审查</td></tr>
<tr><td>审查内容</td><td colspan="3">1、实验动物伦理审查申请表
2、研究方案（版本号：1.0，版本日期：2024年04月22日）
3.1、科学性审查证明文件
3.2、立项证明1
3.3、立项证明2
3.4、科学性审查证明文件
4、研究承诺函
5、培训证书
6、授权书（版本号：1.0，版本日期：2024年01月17日）
7、实验动物伦理审查申请表签字扫描版</td></tr>
<tr><td rowspan="4">审查意见</td><td colspan="3">1. 根据《实验动物管理条例》（2017年修订版）、《关于善待实验动物指导性意见》（2006年）、《实验动物 福利伦理审查指南》（GB/T 35892-2018）和《实验动物 动物实验通用要求》（GB/T35823-2018）等规定和要求，经本伦理委员会审查，本项目符合实验动物伦理的要求，意见如下：
■批准　□修改后批准　□修改后重审
□不批准　□终止或暂停已经开展的动物实验
2.《伦理审核批准文件》是唯一的项目通过伦理审查的证明文件；
3.《伦理审核批准文件》原件一式二份，研究者一份，伦理委员会一份；</td></tr>
<tr><td>年度定期/跟踪审查频率</td><td colspan="2">12个月</td></tr>
<tr><td>批件有效期</td><td colspan="2">2024-05-22 ~ 2025-05-21</td></tr>
<tr><td colspan="3">主任委员（签名）：张国良
深圳市第三人民医院实验动物伦理委员会
2024年05月22日</td></tr>
</table>

联系方式：
地址：深圳市龙岗区布吉镇布澜路29号　　　　邮编：518112
电话：18820242267
