# Supplementary material for: A cell-penetrating bispecific antibody suppresses hepatitis B virus replication and secretion
Source: Virus Res. 2025 Jan 31;353:199531. doi: 10.1016/j.virusres.2025.199531 (PMC11841211; doi:10.1016/j.virusres.2025.199531)
Supplement: Supplementary file 2 [file mmc2.docx]

**Supplement Table1 Monoclonal phage ELISA of HBcAg**

|  | 1st monoclonal phage ELISA | | 2nd monoclonal phage ELISA | | 3rd monoclonal phage ELISA | |
| --- | --- | --- | --- | --- | --- | --- |
| Clones | Coating:  HBcAg | No coating  (1.5%BSA+1.5%OVA) | Coating:  HBcAg | No coating  (1.5%BSA+1.5%OVA) | Coating:  HBcAg | No coating  (1.5%BSA+1.5%OVA) |
| 1 | 0.059 | 0.102 | 0.303 | 0.147 | 1.941 | 0.102 |
| 2 | 0.054 | 0.105 | 2.261 | 0.113 | 2.289 | 0.105 |
| 3 | 0.055 | 0.083 | 0.237 | 0.121 | 2.407 | 0.083 |
| 4 | 0.785 | 0.087 | 0.292 | 0.107 | 2.343 | 0.087 |
| 5 | 0.288 | 0.09 | 0.689 | 0.111 | 2.332 | 0.09 |
| 6 | 0.084 | 0.091 | 0.189 | 0.145 | 2.412 | 0.091 |
| 7 | 0.051 | 0.081 | 0.238 | 0.123 | 2.298 | 0.081 |
| 8 | 0.066 | 0.101 | 0.196 | 0.18 | 2.259 | 0.101 |
| 9 | 0.067 | 0.12 | 2.216 | 0.133 | 2.433 | 0.12 |
| 10 | 0.05 | 0.084 | 0.405 | 0.154 | 2.344 | 0.084 |
| 11 | 0.053 | 0.127 | 1.567 | 0.126 | 2.484 | 0.127 |
| 12 | 0.042 | 0.084 | 2.268 | 0.106 | 1.644 | 0.084 |
| 13 | 0.044 | 0.104 | 0.132 | 0.132 | 1.33 | 0.104 |
| 14 | 0.052 | 0.094 | 0.559 | 0.129 | 1.831 | 0.094 |
| 15 | 0.053 | 0.093 | 0.179 | 0.179 | 2.372 | 0.093 |
| 16 | 0.078 | 0.098 | 1.019 | 0.179 | 1.821 | 0.098 |
| 17 | 0.059 | 0.095 | 2.3 | 0.105 | 2.381 | 0.096 |
| 18 | 0.045 | 0.108 | 0.498 | 0.16 | 2.323 | 0.094 |
| 19 | 0.043 | 0.096 | 0.383 | 0.131 | 2.308 | 0.104 |
| 20 | 0.039 | 0.086 | 0.641 | 0.15 | 2.335 | 0.089 |
| 21 | 0.055 | 0.102 | 1.982 | 0.131 | 2.364 | 0.098 |
| 22 | 0.059 | 0.105 | 0.704 | 0.125 | 2.433 | 0.123 |
| 23 | 0.981 | 0.096 | 0.148 | 0.203 | 2.241 | 0.089 |
| 24 | 0.062 | 0.105 | 0.339 | 0.141 | 2.218 | 0.131 |
| 25 | 0.082 | 0.097 | 0.605 | 0.094 | 2.373 | 0.078 |
| 26 | 0.05 | 0.104 | 0.097 | 0.126 | 2.367 | 0.069 |
| 27 | 0.053 | 0.088 | 0.646 | 0.13 | 0.953 | 0.086 |
| 28 | 0.071 | 0.108 | 0.343 | 0.114 | 2.343 | 0.064 |
| 29 | 0.063 | 0.087 | 0.213 | 0.11 | 2.501 | 0.069 |
| 30 | 0.056 | 0.092 | 0.459 | 0.129 | 1.779 | 0.069 |
| 31 | 0.075 | 0.088 | 1.149 | 0.153 | 1.069 | 0.098 |
| 32 | 0.075 | 0.112 | 0.949 | 0.179 | 2.266 | 0.09 |
| 33 | 0.051 | 0.118 | 0.866 | 0.18 | 1.249 | 0.172 |
| 34 | 0.053 | 0.094 | 0.288 | 0.165 | 2.316 | 0.251 |
| 35 | 0.057 | 0.11 | 2.325 | 0.098 | 2.327 | 0.077 |
| 36 | 0.069 | 0.107 | 0.274 | 0.113 | 2.314 | 0.174 |
| 37 | 0.077 | 0.1 | 1.444 | 0.079 | 1.058 | 0.148 |
| 38 | 0.081 | 0.093 | 0.141 | 0.131 | 2.455 | 0.11 |
| 39 | 0.066 | 0.097 | 0.862 | 0.125 | 2.329 | 0.129 |
| 40 | 0.068 | 0.114 | 0.168 | 0.112 | 2.218 | 0.135 |
| 41 | 0.056 | 0.086 | 0.095 | 0.088 | 2.373 | 0.174 |
| 42 | 0.06 | 0.11 | 0.267 | 0.095 | 1.392 | 0.261 |
| 43 | 0.115 | 0.089 | 1.464 | 0.117 | 2.439 | 0.206 |
| 44 | 0.254 | 0.089 | 0.123 | 0.099 | 2.39 | 0.157 |
| 45 | 1.259 | 0.097 | 2.17 | 0.098 | 2.457 | 0.212 |
| 46 | 0.17 | 0.106 | 0.119 | 0.093 | 0.374 | 0.115 |
| 47 | 0.103 | 0.093 | 0.172 | 0.1 | 2.372 | 0.179 |
| 48 | 0.082 | 0.109 | 0.064 | 0.117 | 0.842 | 0.149 |
| 49 | 0.084 | 0.101 | 0.162 | 0.108 | 2.339 | 0.097 |
| 50 | 0.916 | 0.139 | 0.134 | 0.106 | 0.374 | 0.104 |
| 51 | 1.588 | 0.103 | 0.332 | 0.084 | 2.38 | 0.088 |
| 52 | 1.558 | 0.099 | 1.152 | 0.069 | 1.019 | 0.108 |
| 53 | 2.051 | 0.088 | 0.425 | 0.084 | 2.424 | 0.087 |
| 54 | 0.274 | 0.084 | 0.099 | 0.087 | 2.399 | 0.092 |
| 55 | 0.201 | 0.096 | 0.563 | 0.109 | 2.448 | 0.088 |
| 56 | 0.105 | 0.112 | 0.182 | 0.079 | 2.209 | 0.112 |
| 57 | 1.065 | 0.096 | 0.129 | 0.133 | 0.909 | 0.118 |
| 58 | 1.137 | 0.094 | 0.08 | 0.093 | 1.789 | 0.094 |
| 59 | 1.797 | 0.104 | 0.092 | 0.104 | 2.396 | 0.11 |
| 60 | 1.101 | 0.089 | 0.118 | 0.101 | 2.412 | 0.107 |
| 61 | 0.424 | 0.098 | 0.076 | 0.084 | 2.321 | 0.1 |
| 62 | 0.169 | 0.123 | 0.108 | 0.091 | 0.8 | 0.093 |
| 63 | 0.14 | 0.089 | 0.132 | 0.096 | 2.237 | 0.097 |
| 64 | 0.123 | 0.131 | 0.201 | 0.099 | 2.395 | 0.114 |
| 65 | 0.187 | 0.078 | 0.102 | 0.11 | 1.17 | 0.086 |
| 66 | 0.131 | 0.069 | 0.471 | 0.084 | 2.248 | 0.11 |
| 67 | 0.165 | 0.086 | 0.067 | 0.084 | 2.318 | 0.089 |
| 68 | 0.31 | 0.064 | 0.075 | 0.074 | 2.347 | 0.089 |
| 69 | 0.174 | 0.069 | 0.853 | 0.076 | 2.424 | 0.097 |
| 70 | 0.326 | 0.069 | 0.17 | 0.082 | 2.065 | 0.106 |
| 71 | 0.122 | 0.098 | 0.115 | 0.11 | 2.187 | 0.093 |
| 72 | 0.128 | 0.09 | 0.134 | 0.122 | 0.713 | 0.109 |
| 73 | 0.25 | 0.08 | 0.188 | 0.121 | 1.236 | 0.086 |
| 74 | 0.245 | 0.078 | 0.721 | 0.083 | 2.348 | 0.102 |
| 75 | 0.162 | 0.078 | 0.206 | 0.123 | 2.263 | 0.105 |
| 76 | 1.408 | 0.069 | 0.306 | 0.085 | 2.057 | 0.096 |
| 77 | 0.138 | 0.077 | 0.096 | 0.145 | 2.306 | 0.105 |
| 78 | 0.136 | 0.087 | 0.257 | 0.135 | 2.251 | 0.108 |
| 79 | 0.451 | 0.093 | 0.134 | 0.1 | 0.417 | 0.087 |
| 80 | 0.579 | 0.087 | 0.209 | 0.133 | 2.446 | 0.092 |
| M13KO7 | 0.172 | 0.185 | 0.147 | 0.142 | 0.078 | 0.065 |
| 1%M-PBS | 0.077 | 0.161 | 0.051 | 0.135 | 0.059 | 0.04 |

**Supplement Table2 Molecular Mass of Recombinant Proteins and Antibodies**

| Name | Molecular Weight |
| --- | --- |
| HBcAg | 16.28 kDa |
| preS1 | 13.38 kDa |
| Anti-HBcAg VHH | 13.42 kDa |
| Anti-HBcAg Fc | 77.74 kDa |
| Anti-HBcAg Fc-R9TAT | 81.32 kDa |
| Anti-preS1 Fc | 146.64 kDa |
| Anti-preS1×Anti-HBcAg | 174.56 kDa |
| Anti-preS1×Anti-HBcAg-R9TAT | 183.78 kDa |

**

**

**Supplement figure 1**

We treated HepG2.2.15 with 10μg/mL of the four antibodie, Anti-HBcAg Fc、Anti-HBcAg Fc-R9TAT、Anti-preS1×Anti-HBcAg and Anti-preS1×Anti-HBcAg-R9TAT, for 12 hours respectively, then the cells were washed three times and fixed for immunofluorescence assay. Anti-mouse IgG-FITC was used as detection antibody, DAPI strain for nucleus. We used confocal microscope (ZEISS)to test the immunofluorescence. We perform a quantitative analysis of the obtained microscopic images using Image J software, the mean fluorescence intensity was calculated.

**
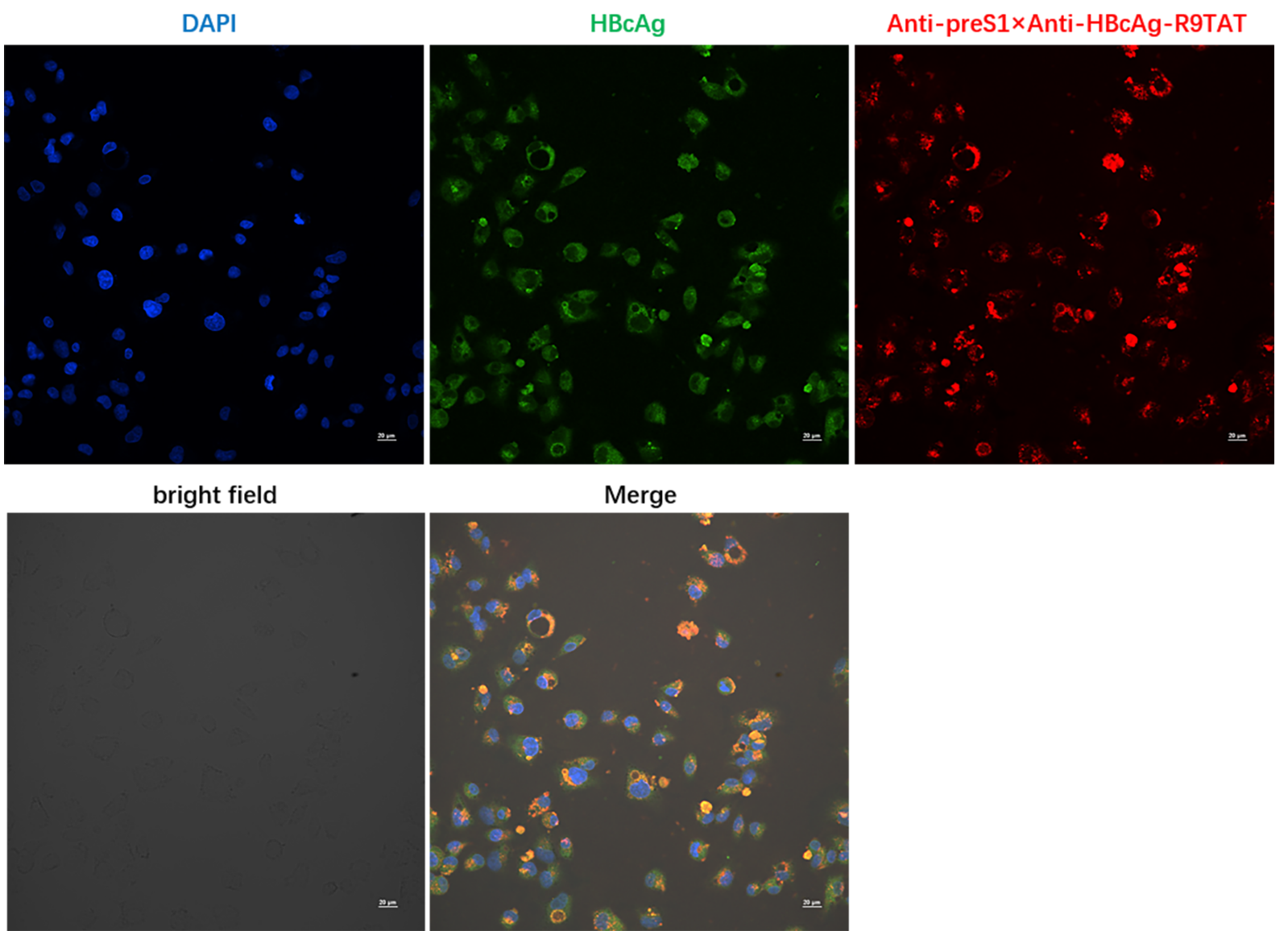
**

**Supplement figure 2**

Immunofluorescence confocal laser scanning microscopy to assess the intracellular localizations of internalized Anti-preS1×Anti-HBcAg-R9TAT and HBcAg. We treated HepG2.2.15 cells with 10μg/mL of Anti-preS1×Anti-HBcAg-R9TAT for 12 hours, then the cells were washed three times and fixed for immunofluorescence assay. Anti-mouse IgG-FITC was used to detect Anti-preS1×Anti-HBcAg-R9TAT, Anti-HBcAg rabbit polyclonal antibodies and Anti-rabbit-AF647 secondary antibodies was used to detect the inner HBcAg, DAPI strain for nucleus.

**
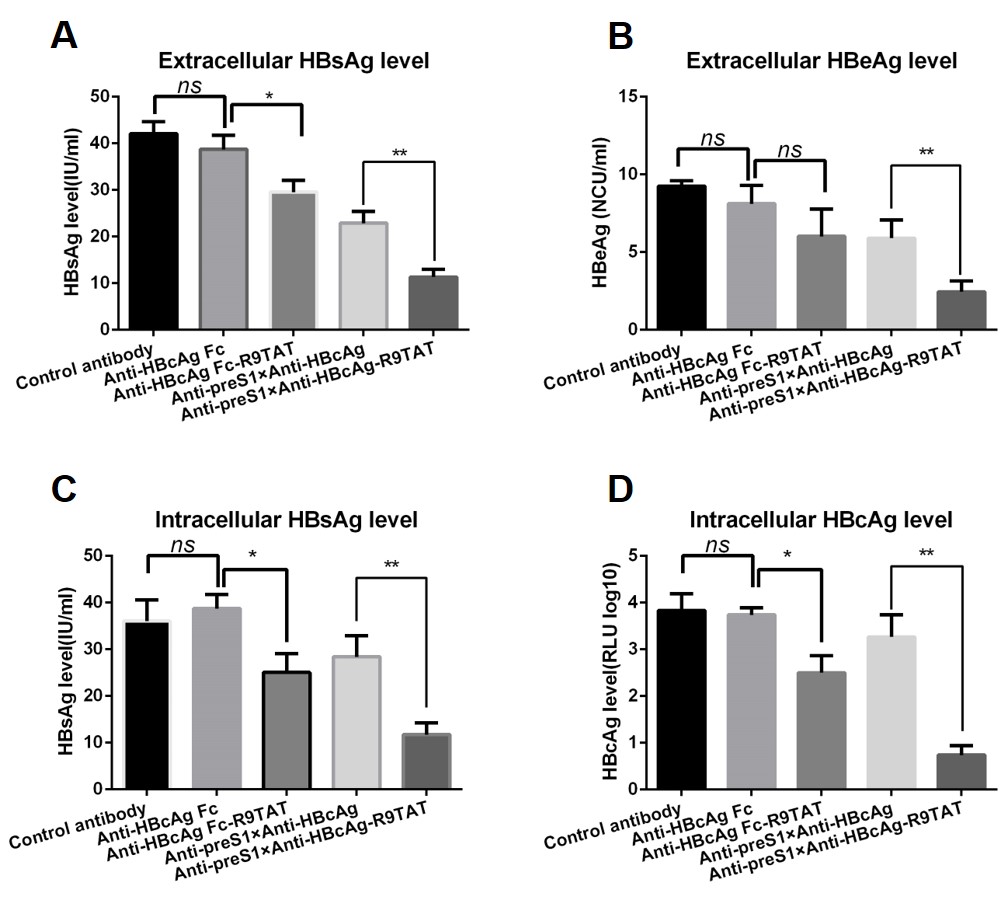
**

**Supplement figure 3**

HepG2.2.15 was treated with 10μg/mL of the five antibodies: Anti-HBcAg Fc、Anti-HBcAg Fc-R9TAT、Anti-preS1×Anti-HBcAg、Anti-preS1×Anti-HBcAg-R9TAT and control antibodies 24 hours respectively. The culture supernatant was collected and tested for extracellular HBsAg(A) and extracellular HBeAg(B) level by ELISA. The cell precipitation was collected and lysis to detect the intracellular HBsAg(C) and HBcAg level(D).

**
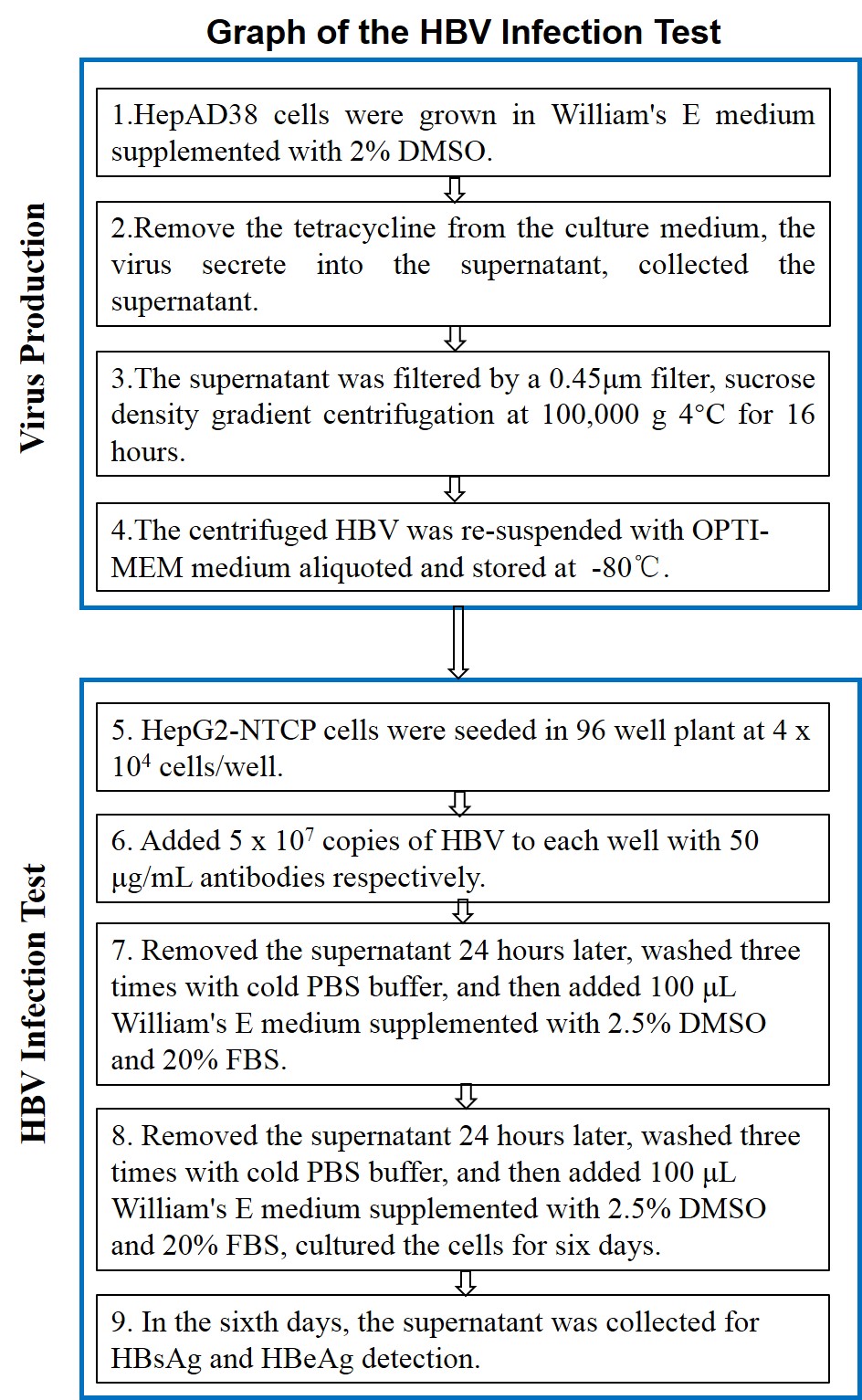
**

**Supplement figure 4**

**
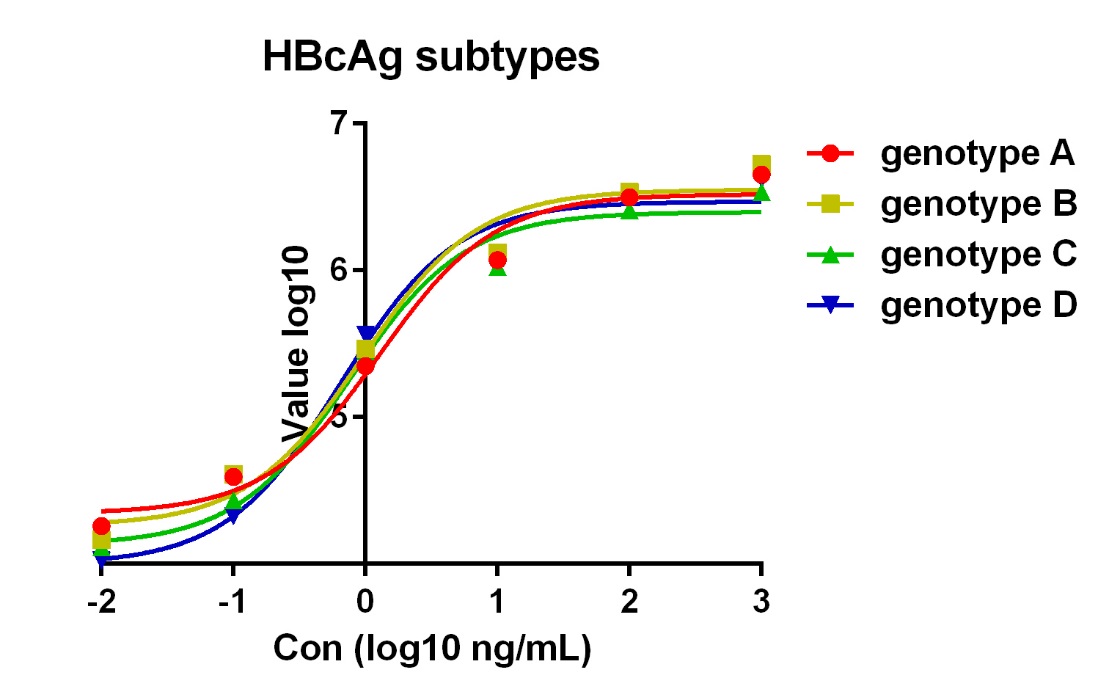
**

**Supplement figure 5**

We tested the binding activity of Anti-HBcAg Fc with HBcAg from subtypes A-D, we found that the Anti-HBcAg Fc could bind to the four different HBcAg subtypes with no significant difference.
